# Comparison between Standardized and Modified EZ-DripLoss Determination Methods in Chicken Breast Meat

**DOI:** 10.3390/ani13061054

**Published:** 2023-03-14

**Authors:** Ana Kaić, Zlatko Janječić, Karla Golub, Klemen Potočnik

**Affiliations:** 1 Department of Animal Science and Technology, University of Zagreb Faculty of Agriculture, Svetošimunska Cesta 25, 10000 Zagreb, Croatia; 2 Department of Animal Nutrition, University of Zagreb Faculty of Agriculture, Svetošimunska Cesta 25, 10000 Zagreb, Croatia; 3 Department of Animal Science, Biotechnical Faculty, University of Ljubljana, Groblje 3, SI-1230 Domžale, Slovenia

**Keywords:** drip loss, meat, poultry, water-holding capacity

## Abstract

**Simple Summary:**

The quality of fresh meat largely relates to water-holding capacity (WHC) which is an important attribute associated with consumer acceptance and food-processing technology. Regarding this, the industry requires methods that can easily, with a great precision, determine the WHC of meat and meat products. The EZ-DripLoss method is relatively new gravimetric method that is used for determination of excessive drip in meat. Currently, there is diversity in the literature regarding the use of EZ-DripLoss methodology. Therefore, this technical note aimed to research drip loss in chicken breast meat measured across the period of three days using two different EZ-DripLoss methodologies. In the standardized EZ-DripLoss method, drip loss is calculated by weighing specialized EZ containers, whereas in the modified EZ-DripLoss method drip loss is calculated by weighing samples. This technical note indicates that different EZ-DripLoss methodology results in different drip loss values in chicken breast meat. Therefore, comparisons of the EZ-DripLoss results should be performed with great caution.

**Abstract:**

The EZ-DripLoss method is relatively new gravimetric method that is used for the determination of excessive drip in meat. The literature reports diversity regarding the use of EZ-DripLoss methodology. In the standardized EZ-DripLoss method, drip loss is calculated as the change in the container weight, whereas in the modified EZ-DripLoss method, it is calculated as the change in sample weight. This technical note aimed to research the relationship between these two methods on chicken breast meat (40 broilers from the line Ross 308) during the measurement interval of 24, 48, and 72 h. The results showed statistically a significant positive linear increase in drip loss values regardless of the used method during all of the investigated measurement intervals. At 24, 48, and 72 h of storage, the average drip loss in the weighed samples was 0.77, 1.40, and 2.23 percentage points greater than in the not weighed samples (*p* < 0.0001), respectively. A strong and positive estimate of correlation coefficients between the drip loss of weighed and not weighed samples was found after 24 h (r = 0.95), 48 h (r = 0.92), and 72 h (r = 0.86). This technical report indicates that the used EZ-DripLoss methodology highly influences the drip loss in chicken breast meat and the comparisons of the EZ-DripLoss results should be performed with great caution.

## 1. Introduction

The quality of fresh poultry meat relates largely to water-holding capacity (WHC) which is an important attribute that is associated with consumer acceptance and food-processing technology [1]. For consumers, poor WHC of meat results in diminished visual appeal due to excess of drip loss, whereas for industry it results in economic losses (reduction of production weight, the loss of moisture, valuable water-soluble proteins and vitamins), and in lower processing characteristics (inferior yields, and lower quality of processed meat) [1,2]. Regarding this, the industry requires methods that can easily and precisely determine the WHC of meat and meat products [3].

In practice, many diverse methods have been used to measure WHC (drip loss, cooking loss, thawing loss, centrifuge force, etc.) and within each methodological approach, several modifications exist. Despite of the modifications, the principles within various methods are generally the same, i.e., a meat sample is weighed before and after a certain treatment (centrifugation, cooking, freezing, etc.) and then the result is expressed as the weight difference in comparison to the initial sample weight. According to Honikel [4], WHC methods can be divided into three basic groups. The first WHC determination group consists of gravimetric methods (bag method, EZ-DripLoss method) where no external force is applied and measures fluid that is lost from fresh meat with gravity as the only force that is exerted on the meat. The second group of WHC methods involves the use of mechanical pressure, where the WHC of the meat can be determined within a few minutes or an hour (centrifugation methods or filter paper press methods). The third group of WHC methods involves application of thermal force, and measures ‘cooking loss’ as meat is usually consumed after thermal treatment [4]. Aside of the methodological factors influencing WHC (anatomical location, fiber orientation, sample weight and geometry, external conditions, etc.), the most influential factor when measuring WHC is the type of treatment/methodology that is applied [1].

The EZ-DripLoss method is a relatively new gravimetric method for the determination of excessive drip in meat that is widely used in the industry. The EZ-DripLoss method was first introduced by Rasmussen and Anderson [5] and then described in detail in “Instruction manual for EZ-DripLoss” [6]. In brief, the EZ-DripLoss is gravimetric method in which the standardized meat sample (25 mm height × 25 mm diameter) is removed with circular knife, and then is left in a specialized EZ container for a 24 h long period to drip [5]. The literature confirms that the EZ-DripLoss method has a high sensitivity, is easier to perform in a reproducible way, uses less space than the other conventional methods, and is easy to handle under abattoir conditions [3,7,8,9]. However, it must be taken into consideration that there is a diversity in the literature regarding the use of EZ-DripLoss methodology.

The standardized EZ-DripLoss method was first presented as a procedure where muscle samples are not weighed before being placed in the containers [6]. This methodology implies the necessity of weighing an empty container, the container with a meat sample and drip loss, and that same container only with drip loss, after removing the meat sample [6]. Drip loss assessment using the standardized EZ-DripLoss methodology has been investigated in pork [3,10], beef [11], and deer meat [12].

However, the literature reports that EZ-DripLoss assessment in numerous studies is based on the weighing of the meat samples before storage, and drip loss is calculated as change in the sample weight. This methodology, where the EZ-DripLoss is evaluated by weighing samples, was investigated in pork [3,10], lamb [13], mutton [14], poultry [15], alpaca [9], and horse meat [16].

In addition to inconsistencies of the used methodology, it is well known that the results of the drip loss methods used are highly dependent on the area sampled, its weight, the fiber direction of the sample, and the storage period [3,13,17].

Due to the variations in the used methodology, it is questionable if the drip loss values that are obtained by different sample handling techniques (weighed samples vs. not weighed samples) are comparable between studies. Namely, in the standardized method, developed by Danish Meat Research Institute [6], samples are not dabbed before final weighing, i.e., the sample is kept in the pre-weighed container and weighed with it. According to Correa et al. [10], the absence of dabbing affects the reliability of the measurement and may lead to underestimation of the drip that is excluded from the sample during storage. Furthermore, according to this methodology, drip loss is measured after 24 h of storage time. However, Honikel [4] recommended a longer storage time for drip loss assessment, independent from the used method. This inconsistency in the two sample handling techniques within the EZ-DripLoss procedure was further investigated in pork meat by Filho et al. [3] and Correa et al. [10]. Filho et al. [3] reported that sample dabbing does not improve the reliability of the EZ-DripLoss methodology (weighed vs. not weighed samples) for the drip loss assessment and overall pork quality evaluation, and that the accuracy of the method could be increased by using a longer storage time (from 24 h to 48 h). They also pointed out that the EZ-DripLoss method of not weighed samples could distinguish drip loss into meat-quality categories and thus recommended this method for meat categorization. Correa et al. [10] also agreed that longer storage time should be considered (for 48 h) when drip loss is evaluated by using EZ-DripLoss method. However, to increase the accuracy of the EZ-DripLoss method and other pork quality attributes Correa et al. [3] recommended sample dabbing and weighing prior to drip loss evaluation.

Furthermore, there is a deficiency in our knowledge of the EZ-DripLoss method’s suitability for poultry meat analysis. To our knowledge, with poultry meat, there are only a few studies where the drip loss was evaluated by using the EZ-DripLoss method [15,16]. Therefore, the aim of this technical note was to investigate the relationship between two methods (weighed samples vs. not weighed samples) for reliable EZ-DripLoss assessment in chicken breast meat.

## 2. Materials and Methods

### 2.1. Animals, Slaughter, and Handling Procedure

The report was conducted on chicken breast meat samples originating from 40 broilers from the line Ross 308. The animals were handled according to Croatian legislation (Animal Protection Act, Official Gazette 102/17; Regulation on the protection of animals used for scientific purposes, Official Gazette 55/13), and approved by the Bioethical committee for the protection and welfare of animals at the University of Zagreb, the Faculty of Agriculture, Croatia (Class: 114-04/20-03/10; Ref. 251-71-29-02/19-20-2). The slaughter of the animals took place at the age of 35 d. After the slaughtering procedure, the carcasses were eviscerated, weighed (average slaughter weight = 1.765 ± 0.180 kg), and chilled at 4 °C for 24 h.

### 2.2. EZ-DripLoss Sampling

The EZ-DripLoss determination was performed on the samples that were taken from the pure fresh chicken breast (n = 40) with the average weight of 525.45 ± 0.180 g. Muscle samples for the analyses were taken from the cranial edge of the *pectoralis* muscle of each breast at 24 h *post mortem*. The EZ-DripLoss determination was performed by cutting two slices of 2.5 cm thickness from which cylindrical muscle cores were taken in duplicate (using an EZ-DripLoss circular knife; n = 160). The muscle cores were removed following vertical fiber orientation, and were 2.5 cm in diameter [5].

There were two different methodologies for the EZ-DripLoss measurement that were applied. In the first one (standardized; not weighed), muscle cores were not weighed before placing in the special pre-weighed EZ containers (Danish Meat Research Institute, Taastrup, DEN). After the measurement intervals of 24, 48, and 72 h, each container was weighed while including the muscle core and drip loss, and once again after removing the muscle core, only for drip loss [6]. In the second methodology (modified; weighed), the muscle cores were weighed before storage ($\bar{x}$ = 7.6 g), and once again after each measurement interval (24, 48, and 72 h). Before each sample weighing, as suggested Correa et al. [10], the muscle core surface of all the weighed samples was gently dabbed with a paper towel.

During the experiment, all the samples were placed within the same refrigerator at an average temperature of +3.5 °C (±0.80 s.d.) and average humidity of 85% (±2.70 s.d.).

The EZ assessment of the not weighed samples (standardized method; EZ_S_) was performed according to the Danish Meat Research Institute [6]:

$${\mathrm{EZ}}_{s}=[{(W}_{I}-W_{c})/{(W}_{t}-W_{c})]\times 100$$

where:

$W_{c}$ = weight of the empty EZ-DripLoss container
$W_{t}$ = weight of the EZ-DripLoss container with the meat sample and drip loss, and
$W_{I}$ = weight of the EZ-DripLoss container with drip loss.

The EZ assessment of the weighed samples (modified method; EZ_M_) was performed as the change in the sample weight as follows:

$${\mathrm{EZ}}_{M}=[{(W}_{I}-W_{f})/W_{f}]\times 100$$

where:

$W_{I}$ = initial weight of the sample, and
$W_{f}$ = final weight of the sample.

As introduced by Rasmussen and Andersson [5], the mean value of muscle cores taken in duplicate was used for each EZ-DripLoss assessment.

### 2.3. Statistical Analyses

The data were analyzed using the SAS/STAT software package version 9.4 [18]. Descriptive statistic parameters for drip loss were calculated using the MEANS procedure, while the effect of different methods on drip loss was evaluated with a paired *t*-test using the TTEST procedure. The differences were significant if *p* < 0.05. The relationship between the drip loss of the weighed and not weighed samples was determined by estimation correlation coefficients using the CORR procedure.

## 3. Results and Discussion

### 3.1. Variations of Drip Loss Values in Relation to Different Measurement Intervals within and between Weighed and Not Weighed Samples

Distributions of drip loss values that were obtained from the weighed and not weighed samples using EZ-DripLoss methods (EZ_S_ vs. EZ_M_) after 24, 48, and 72 h in chicken breast meat are shown in Table 1. 

The average drip losses that were determined in the not weighed samples were 0.95% within 24 h, 1.30% within 48 h, and 1.38% within 72 h, whereas the weighed samples were 1.72% within 24 h, 2.70% within 48 h, and 3.62% within 72 h. Although there is considerable variability in WHC between animals, the following comparison with different species should give us deeper insight into the drip loss values that were obtained by different EZ-DripLoss methodologies. Greater average drip loss values of not weighed samples were found with beef [11] and deer meat [12] than the ones of the present technical report. Mergeduš et al. [11] found that the average drip loss of meat from bulls that were fattened under commercial conditions was 1.40% within 48 h of storage period. Razmaitė et al. [12] found that the average drip loss in meat from free-living red deer was 1.80% within 24 h, and from farmed red deer, it was 3.42% within 24 h.

Correa et al. [10] and Filho et al. [3] compared two sample handling techniques (weighed vs. not weighed samples) within the EZ-DripLoss procedure. Similar to the present report, Correa et al. [10] and Filho et al. [3] found greater average drip losses of weighed and not weighed pork samples after 24 and 48 h of storage period. The average drip loss of the weighed pork samples that were reported by Correa et al. [10] was 5.40% within 24 h and 6.36% within 48 h, while for the not weighed samples drip loss was 3.54% within 24 h and 4.66% within 48 h. The average drip loss of the weighed pork samples reported by Filho et al. [3] was 3.13% within 24 h, and 5.19% within 48%, while for the not weighed samples drip loss was 3.10% within 24 h and 4.40% within 48 h.

With the chicken breast meat, Graberec et al. [15] found greater average drip loss values of weighed samples after 24 and 48 h of storage period than the ones of the present report. Graberec et al. [15] found that the average drip loss of breast meat from commercial chicken hybrids Ross 308 was 2.38% within 24 h and 2.74% within 48 h. However, Graberec et al. [15] reported greater coefficients of variation for drip loss of weighed samples for 24 h (85.36%) and for 48 h (75.51%) than in our report (Table 1). They explained that this greater variability within the samples could be due to the pre-slaughter conditions, post-slaughter factors affecting meat quality, and complex manipulative procedures with the samples (dabbing, weighing, operator handling) in their trial.

On the contrary, lower average drip loss values of weighed samples were found with lamb [13], mutton [14], and meat from adult horses [16] than the ones of the present report. Namely, Kaić et al. [14] aimed to assess drip loss measurements in mutton that were taken by EZ-DripLoss and bag methods, and reported average drip losses of 0.65% within 24 h and 0.93% within 48 h. Holman et al. [13] investigated the effect of muscle fiber orientation and measurement interval on lamb meat drip losses using the EZ-DripLoss method, and found average drip losses of 0.41% within 24 h, 0.67% within 48 h, 0.96% within 72 h, and 1.49% within 96 h. Razmaitė et al. [16] aimed to assess the influence of gender, age, and carcass weight on the properties of meat from adult obsolescent horses. They found that the average drip losses for mares was 1.22%, for stallions was 0.77%, and for geldings was 0.43% within 24 h Compared to our results, lower drip loss values of weighed samples that were found in mutton, lamb, and horse meat could be related to the numerous properties that affect WHC and depend on many interrelated factors, so it is difficult to explain when considering each of them independently. However, deeper insight into the studies could suggest that these lower values could be related to the chemical composition of the investigated muscles. Namely, it is expected that muscles with a lower fat content (such as m. *pectoralis* in the present study) tend to have greater drip loss. Lawrie [19] suggested that lower intramuscular fat content in the muscles tightens the microstructure of meat, and with less incorporated water within the structure causes lower WHC, i.e., greater drip loss. The results of the present technical note showed a positive linear increase in drip loss values regardless of the used method during all of the investigated measurement intervals (24, 48, and 72 h). The differences in the mean values for drip loss of the not weighed samples between 24 and 48 h was 0.35%, and between 48 and 72 h was 0.08%, respectively. The differences in the mean values for drip loss of the weighed samples between 24 and 48 h was 0.98%, and between 48 and 72 h was 0.92%, respectively. This is in accordance with previous studies using the EZ-DripLoss method [3,8,12,13,15,16,20]. Namely, it has been established that the exudation is a slow process that lasts for days, during which water is expelled from the myofibrillar lattice, accumulated in the extracellular space, and then progressively drained out of the muscle as drip loss [15,16].

All the coefficients of variation of the not weighed samples (72.83–88.51%) were greater than that of the weighed samples (40.12–64.39%; Table 1). However, the variability within the weighed samples was greater than of the not weighed samples (24.27% vs. 15.68%). This larger variation within the weighed samples could be related to the more complex manipulative procedures with these samples (dabbing, weighing). Contrary to our results, Correa et al. [10] and Filho et al. [3] found higher coefficients of variation for weighed samples than for the not weighed samples in pork meat. Correa et al. [10] reported coefficients between 42.11% and 51.77% for not weighed samples, and between 38.58% and 43.93% for weighed samples, whereas Filho et al. [3] reported coefficients between 47.89% and 58.71% for not weighed samples, and between 49.45% and 63.95 % for weighed samples.

The results showed a significant difference in drip loss determined by weighed and not weighed samples (EZ_S_ vs. EZ_M_) at the same measurement interval (*p* < 0.0001; Table 2).

At 24, 48, and 72 h of storage the average drip loss in weighed samples was greater for 0.77, 1.40, and 2.23 percentage points than in the not weighed samples. With pork meat, Correa et al. [10] also confirmed greater average drip loss in the weighed samples than in the not weighed samples after 24 h (for 1.86 percentage points) and 48 h (for 1.70 percentage points) of storage. They stated that that these differences are due to the dabbing procedure which helped to remove the exudate that is present on the core surface. This affects the measurement reliability because the weight of the container with exudate does not consider any remaining drip loss that is exuded from the sample during storage. However, Filho et al. [3] found this true only for drip loss that was measured after 48 h of storage in pork samples. According to our experience, besides the dabbing effect, differences could also be explained with greater manipulative procedures related to sample weighing, i.e., weighing the samples before placing them on the containers and re-weighing the samples at the end of each measurement interval. Furthermore, Filho et al. [3] explained that differences in drip loss values between the EZ-DripLoss methods could be related to different water loss rates between the studies. With pork meat, Filho et al. [3] found that about 65% of the drip loss occurred in the first 24 h of storage (60% for weighed samples, and 70% for not weighed samples), whereas Correa et al. [10] found that about 80% of the drip loss occurred in the first 24 h of storage (85% for weighed samples, and 76% for not weighed samples). As was found by Filho et al. [3], the results of the present technical note revealed similar differences in the water loss rate in the first 24 h of storage. Within our samples, about 68% of the drip loss occurred in the first 24 h of storage (63.7% for the weighed samples and 73% for the not weighed samples). Contrary to Filho et al. [3], the analysis of our technical note showed a significant difference in drip loss values between the weighed and not weighed samples (Table 2). Therefore, drip loss evaluation after 24 h may underestimate drip loss of different meats during the storage period, and a longer storage time period should be recommended, regardless of the used EZ-DripLoss method. Similar findings were previously reported in studies using the EZ-DripLoss method [3,8,13,17,20]. With pork meat, Otto et al. [8] and Filho et al. [3] recommend using a 2-day measurement interval to determine drip loss. With beef meat, drip loss should be evaluated using a 3-day measurement interval [20], whereas with lamb meat [13] and chicken meat [15] using longer measurement intervals is recommended (more than 4-days).

### 3.2. Correlations between the Drip Loss of the Weighed and Not Weighed Samples

The results in Table 3 confirm a very strong positive estimate of correlation coefficients (*p* < 0.0001) in drip loss of the weighed samples and drip loss of the not weighed samples during the 24, 48, and 72 h of storage period.

These results are in general agreement with previously explained theoretical expectations on this issue [21,22] during which water that is expelled from the myofibrils accumulates in the muscle, and over time is drained out of the muscle. In mutton, Kaić et al. [14] confirmed high correlations between drip loss by weighing samples after 24 and 48 h of storage (r = 0.93). They stated that these strong positive correlation coefficients are reasonable, due to the repeated measurements of the same samples. In pork meat, Filho et al. [3] also found high correlations between drip loss by weighing samples after 24 and 48 h of storage (r = 0.96), and between drip loss by weighing containers after 24 and 48 h of storage (r = 0.97).

The results of the present technical note also showed visible strong positive correlations between drip loss of the weighed and not weighed samples determined after 24 h (r = 0.95), 48 h (r = 0.92), and 72 h (r = 0.86), respectively. Therefore, the results suggest that standardized and modified EZ-DripLoss methods in chicken meat provide similar results for measuring drip loss. Our results are in accordance with Filho et al. [3] who found high correlations between drip loss by weighing the samples and drip loss by weighing the containers after 24 h (r = 0.97) and 48 h (r = 0.95) of storage in pork meat. In addition, the results of the present technical note indicate that despite of a strong positive relationship, the correlation coefficients between our EZ-DripLoss methods during the measurement interval are slightly lower (Table 3).

## 4. Conclusions

The technical note indicates that drip loss in chicken breast meat is highly dependent on the used EZ-DripLoss methodology, i.e., assessment based on the change in sample weight loss (modified EZ-DripLoss method) or container weight loss (standard EZ-DripLoss method). Although correlation coefficients between the used methods are strong and positive, indicating that they provide similar results for measuring drip loss for chicken meat, paired t-test analysis revealed significant differences. Namely, it could be expected that studies using the modified EZ-DripLoss method have a greater average drip loss than using the standardized EZ-Driploss method. Regardless of the used EZ-DripLoss method, the measurement interval showed a positive linear relationship with drip loss, indicating that a longer period of storage time is needed for its stabilization and should be reported in studies. Comparisons of the EZ-DripLoss results that were obtained with different methodologies should be performed with great caution.

## Figures and Tables

**Table 1:** Means ($\bar{x}$) with standard deviation (SD), coefficients of variation (CV), minimum (Min), and maximum (Max) for the drip loss of chicken breast meat in relation to standardized (EZS; not weighed samples) and modified (EZM; weighed samples) EZ-DripLoss methodologies and different measurement intervals (24, 48, and 72 h).

Attribute/Measurement Interval		$\bar{x}$	SD	CV, %	Min	Max
	24 h	0.95	0.840	88.51	0.00	3.84
EZ_S,_^%^	48 h	1.30	0.917	70.50	0.13	3.83
	72 h	1.38	1.003	72.83	0.27	4.26
EZ_M,_^%^	24 h	1.72	1.105	64.39	0.40	4.76
	48 h	2.70	1.176	43.52	0.90	5.45
	72 h	3.62	1.451	40.12	1.20	7.20

**Table 2:** Paired *t*-test analysis for the drip loss of chicken breast meat in relation to standardized (EZ_S_; not weighed samples) and modified (EZ_M_; weighed samples) EZ-DripLoss methodologies and different measurement intervals (24, 48, and 72 h).

Attribute	$\bar{x}$	DF	*t*-Value	*p*-Value	Sig.
EZ_M_24_–EZ_S_24,_^%^	0.776	39	12.15	<0.0001	***
EZ_M_48_–EZ_S_48,_^%^	1.400	39	18.10	<0.0001	***
EZ_M_72_–EZ_S_72,_^%^	2.237	39	18.35	<0.0001	***

$\bar{x}$: mean of the paired measurements; DF: degrees of freedom; Sig.: level of significance, *** *p* < 0.0001.

**Table 3:** Correlations between standardized (EZ_S_; not weighed samples) and modified (EZ_M_; weighed samples) EZ-DripLoss methods in chicken breast meat determined after 24, 48, and 72 h.

	EZ_M_24_	EZs__24_	EZ_M_48_	EZs__48_	EZ_M_72_	EZs__72_
EZ_M_24_	1.00					
EZ_S_24_	0.950 ***	1.00				
EZ_M_48_	0.890 ***	0.793 ***	1.00			
EZ_S_48_	0.921 ***	0.905 ***	0.920 ***	1.00		
EZ_M_72_	0.736 ***	0.622 ***	0.946 ***	0.838 ***	1.00	
EZ_S_72_	0.840 ***	0.836 ***	0.906 ***	0.954 ***	0.864 ***	1.00

Level of significance: *** *p* < 0.0001.

## Data Availability

The data that support the findings of this technical note are available from the corresponding author, A.K., upon reasonable request.
